# Severe silent ischaemia detected with an Apple Watch in the home setting: a case report

**DOI:** 10.1093/ehjcr/ytae043

**Published:** 2024-01-30

**Authors:** Rudolph W Koster, Robbert J de Winter, Hein J Verberne, Anje M Spijkerboer, Steven A Chamuleau

**Affiliations:** Amsterdam UMC Heart Center, Department of Cardiology, Amsterdam Cardiovascular Sciences, Amsterdam UMC, Location Academic Medical Center, Meibergdreef 9, 1105 AZ Amsterdam, The Netherlands; Amsterdam UMC Heart Center, Department of Cardiology, Amsterdam Cardiovascular Sciences, Amsterdam UMC, Location Academic Medical Center, Meibergdreef 9, 1105 AZ Amsterdam, The Netherlands; Department of Radiology and Nuclear Medicine, Amsterdam UMC, Location Academic Medical Center, Meibergdreef 9, 1105 AZ Amsterdam, The Netherlands; Department of Radiology and Nuclear Medicine, Amsterdam UMC, Location Academic Medical Center, Meibergdreef 9, 1105 AZ Amsterdam, The Netherlands; Amsterdam UMC Heart Center, Department of Cardiology, Amsterdam Cardiovascular Sciences, Amsterdam UMC, Location Academic Medical Center, Meibergdreef 9, 1105 AZ Amsterdam, The Netherlands

**Keywords:** Myocardial ischaemia, Silent ischaemia, Ischaemia detection, Apple Watch, Case report

## Abstract

**Background:**

The Apple Watch has the capability to record a lead 1 electrocardiogram (ECG) and can identify and report atrial fibrillation. The use for detecting myocardial ischaemia is not endorsed by Apple but is documented in this case.

**Case summary:**

A 76-year-old man made a lead 1 ECG with his Apple Watch immediately after exercising on a cross trainer. He was fully asymptomatic. The ECG showed an unusual negative T-wave in this lead 1 that deepened in a few minutes and returned to normal after 22 min. He consulted a cardiologist and a standard exercise ECG confirmed the negative T-wave in lead 1 after maximal exercise and in addition showed widespread ST-depression indicating myocardial ischaemia, again without any clinical symptoms. Further studies revealed severe obstructive three-vessel coronary artery disease that was considered not suitable for percutaneous intervention. A coronary artery bypass operation on all involved vessels was performed successfully. Recovery was uneventful and an exercise ECG repeated 11 weeks later was normal.

**Discussion:**

We demonstrated that the lead 1 ECG made with the Apple Watch can reliably record T-wave changes indicating myocardial ischaemia. The use of the Apple Watch to document ischaemic changes should be studied systematically for its potential to identify myocardial ischaemia, mainly triggered by symptoms but maybe for asymptomatic persons as well.

**Learning points**

- The single lead 1 electrocardiogram as recorded by the Apple Watch allows diagnostic interpretation of ischaemic changes that can trigger further diagnostic action if indicated.
- Extensive exercise-induced reversible myocardial ischaemia may remain asymptomatic and can result in exercise-induced sudden death.

## Introduction

The Apple Watch offers an electrocardiogram (ECG), derived from the back of the watch on the one arm and the index finger from the other arm on the crown, which results in a single lead 1 ECG. This lead 1 ECG can detect atrial fibrillation and other arrhythmias, a capability that is supported by Apple.^1–3^ While the frequency characteristics of the lead are not described in the documentation, the high-frequency QRS complex and the low-frequency ST segment and T-wave seem to be accurately displayed.^4^ Apple does not recommend to use the Apple Watch for the detection of myocardial infarction or ischaemia.

## Summary figure

Timeline of the diagnostic process until the decision to perform coronary bypass surgery.

iFR, instantaneous wave-free ratio.

**Figure:**
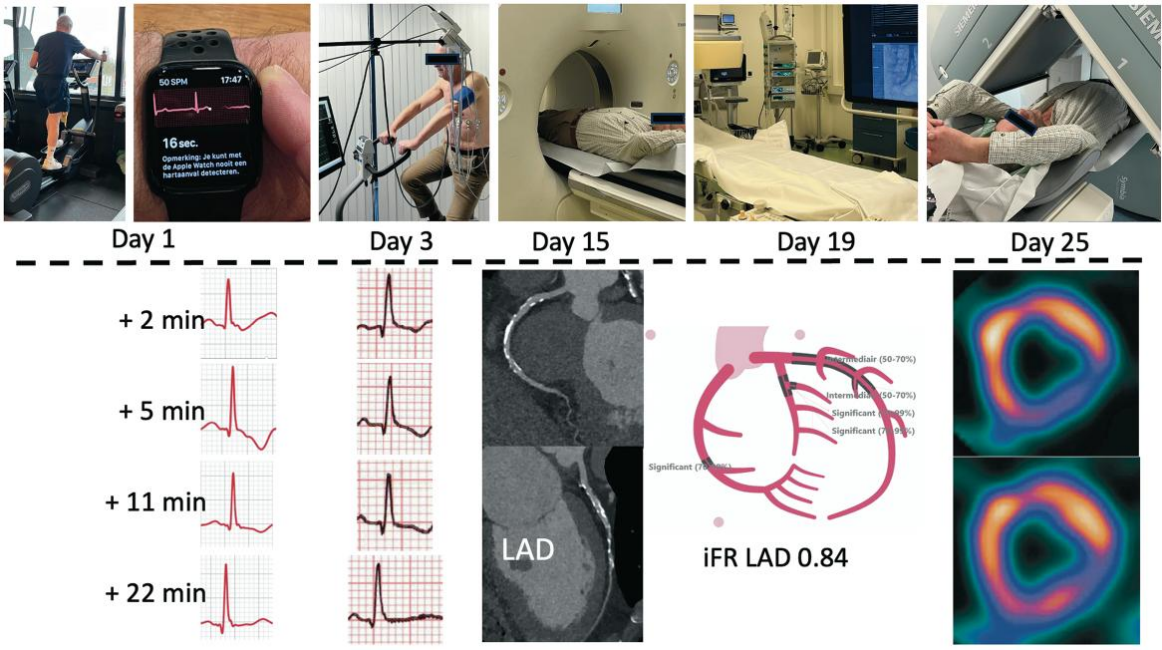


## Case presentation

**Figure 1:**
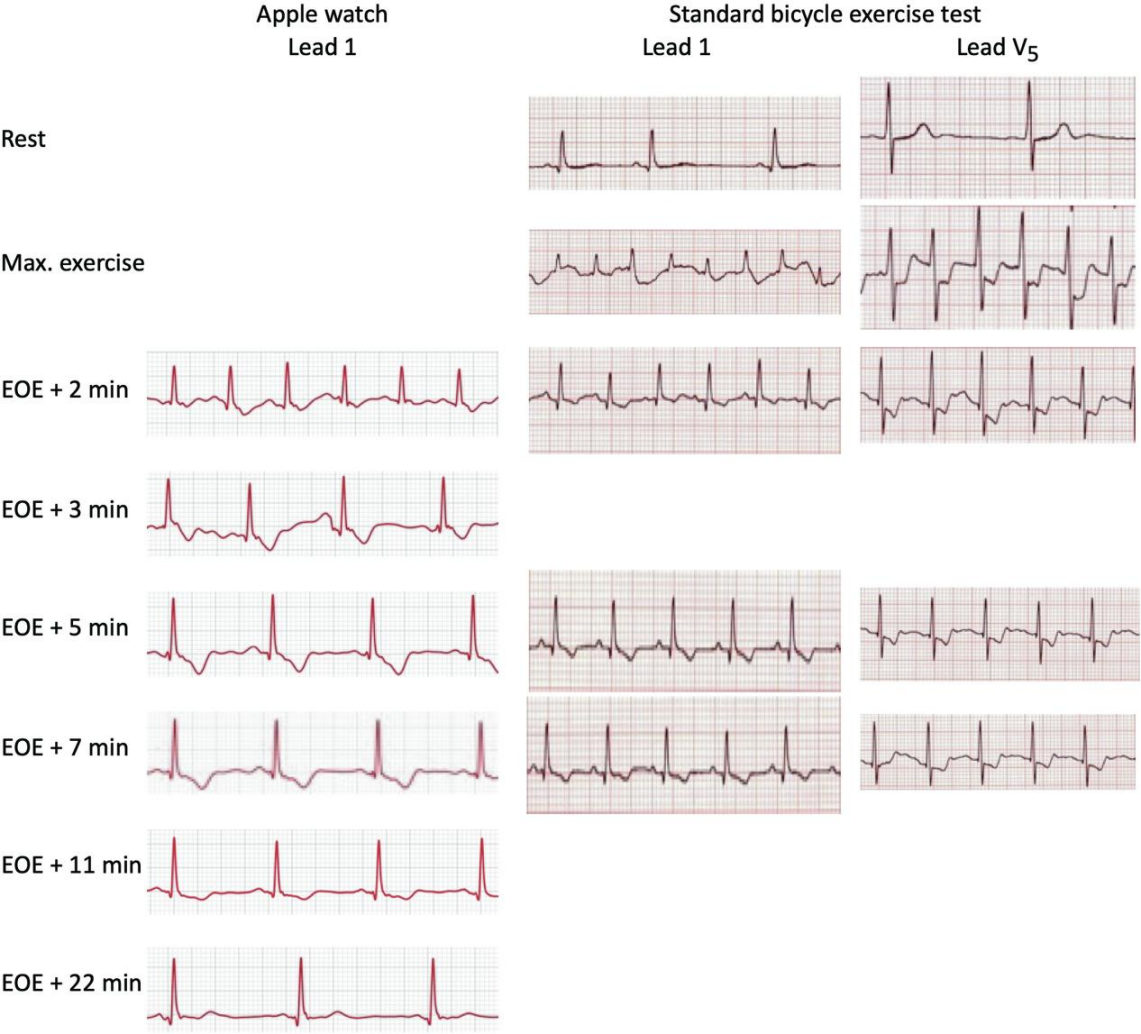
Single lead 1 recording from the Apple Watch (left panel) recorded from 2 min after the end of exercise (EOE +2) on a cross trainer. A standard exercise test performed 2 days later (right panel) shows lead 1 and lead V_5_ prior to exercise, at peak exercise, and at post-exercise moments that match the recording of the Apple Watch. EOE, end of exercise.

A 76-year-old asymptomatic man used to exercise weekly at a local gym. Among others, he used a cross trainer, usually exercising for 10 min with a maximum heart rate of 150/min. Very infrequently, immediately after the exercise, he would activate his Apple Watch and make a lead 1 ECG, solely out of interest. He never noticed any abnormality, except an occasional premature ventricular complex (PVC). During the last ECG recording, he noticed immediately after exercise, a negative T-wave that became more prominent in ∼7 min, then slowly returned back to a normal positive T-wave after 22 min (*Figure 1*). Before, during, and after the episode, he was completely asymptomatic for chest pain, shortness of breath, dizziness, or palpitations.

The family history was strongly positive for coronary artery disease (CAD) with angina pectoris and myocardial infarction. He was known with elevated cholesterol for >30 years and with diabetes mellitus Type 2 for 4 years. He never smoked and had a normal blood pressure. He was treated with statins for 30 years reducing the LDL-cholesterol to <2.3 mmol/L (with atorvastatin 40 mg) and with metformin 500 mg twice daily.

He consulted a cardiologist and there was agreement that this Apple Watch observation warranted further investigation. A standard ECG was completely normal, as was physical examination. A bicycle exercise test was performed, where the patient exercised 16 min with a workload up to 205 W. He achieved a maximum heart rate of 169/min and a blood pressure of 226/66 mmHg. Exercise was terminated because of exhaustion, without any symptom suggestive of myocardial ischaemia. The ECG showed sinus tachycardia, occasional PVCs, and extensive precordial and inferior ST-segment depression up to 4 mm in lead V5 (*Figure 1*). The changes disappeared again in ∼10 min.

**Figure 2:**
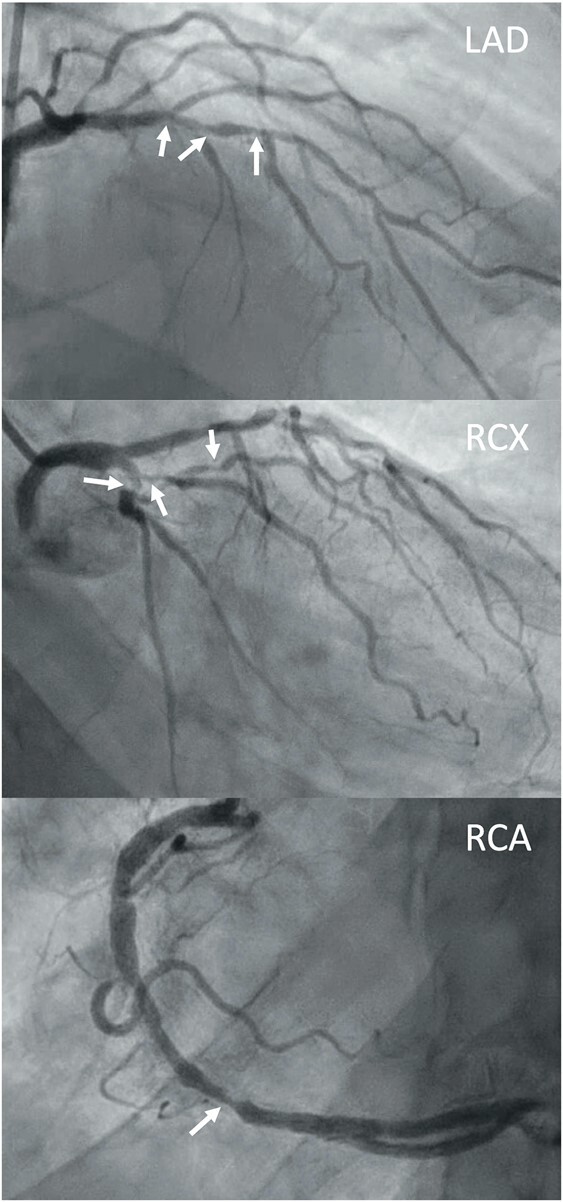
Coronary angiogram, showing severe obstructions in all main vessels, indicated by the arrows. LAD, left anterior descending; RCX, circumflex branch; RCA, right coronary artery.

**Figure 3:**
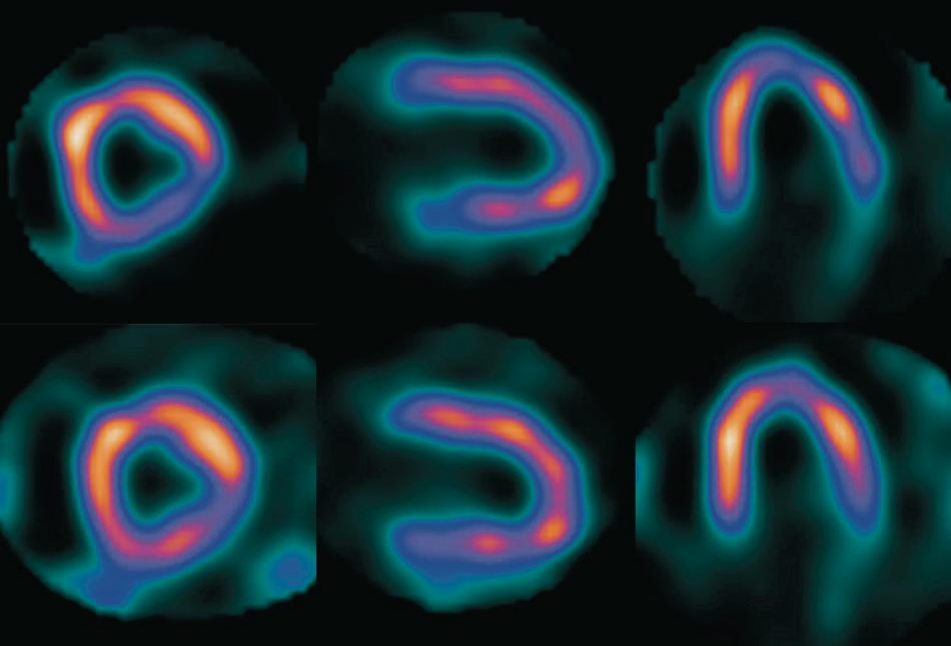
Myocardial perfusion scan post-stress images (top row) in the short axis, vertical long axis, and horizontal axis (respectively, from left to right) compared with myocardial perfusion scan at rest (lower row) indicative of severe inducible myocardial ischaemia.

A transthoracic echocardiogram was normal. A cardiac computed tomography scan revealed stenoses in all coronary arteries (CAD-RADS 4). The severity of the stenoses could not be determined because of extensive calcifications. Additional invasive coronary angiography showed three-vessel disease with significant stenoses in the mid–right coronary artery, a long stenosis in the proximal to mid–left anterior descending (LAD) incorporating the first diagonal (D1) and second diagonal (D2), and in the circumflex branch (RCX) and first marginal branch (MO1) (Modified Dukes score 4) (*Figure 2*). The instantaneous wave-free ratio measured in the LAD was 0.84. An ECG-triggered myocardial perfusion scan with exercise and at rest showed extensive myocardial perfusion abnormalities in the inferior, lateral, and distal anterior wall, involving 16% of the myocardium. There was almost complete reversibility in rest (*Figure 3*). There were no regional wall motion abnormalities. The ejection fraction was 54% at exercise and 57% at rest.

The heart team proposed surgical intervention, taking into account extensive scintigraphic myocardial ischaemia from a severe three-vessel disease that was not well suitable for percutaneous intervention, in conjunction with diabetes.^5^ Also, the silent nature of the documented myocardial ischaemia made symptom-limited exercise or assessment of the effect of anti-anginal medical therapy impossible.

Coronary bypass surgery was performed with four bypasses on all coronary arteries involved (left internal mammary artery–LAD, aorta–D1–MO1–posterior descending branch). Postoperative recovery was uneventful. A repeat-exercise ECG 11 weeks after surgery with a similar exercise level was normal, without any ST-segment alterations.

## Discussion

The presented case is unusual, not only because ischaemia was detected with an Apple Watch in an asymptomatic person, but also because lead I is not ideal for the detection of myocardial ischaemia. Nevertheless, the abnormal and progressive negative T-wave in this lead 1, returning to normal proved an indicator of exercise-induced silent ischaemia. It fully matched the T-wave changes in lead 1 documented in the exercise ECG 2 days later, as shown in *Figure 1*.

The Apple Watch is capable of automatically monitoring the heart rhythm for atrial fibrillation with the photoplethysmography sensor and provides an indication of an irregular heart rhythm with the irregular rhythm notification feature.^1,2^ Such monitoring is not possible for myocardial ischaemia as it requires the initiative of the wearer of the watch to suppress motion when touching the crown during the 30 s of the ECG recording. However, our observation supports the feasibility to instruct the wearer of the watch to make a 30 s lead 1 ECG in case of symptoms of chest pain and send a PDF file of the recording to a treating physician. Even all standard and precordial leads can be recorded, albeit with more effort and required training, but with a fair agreement when compared with a standard 12-lead ECG.^6^ This option is not supported by Apple and the diagnostic value to detect ischaemia has not been sufficiently established, nor the potential negative psychological and societal consequences of false-positive findings. This also includes unwarranted anxiety and the risk of a cascade of investigations without clinical benefit. This case report shows the potential of expanding the use of e-health applications in the home setting to a broader spectrum of cardiovascular diseases, but notably requires rigorous validation before advancing to implementation in routine clinical care.

## Data Availability

The data underlying this article will be shared on reasonable request to the corresponding author.

## Lead author biography

Dr Rudolph W. Koster trained in the Academic Hospital of the University of Amsterdam and became a cardiologist in 1977 where he was a staff member throughout his career. He retired from clinical work in 2016 but continued research in out-of-hospital cardiac arrest until now. The research focuses on resuscitation and the use of the automated external defibrillator, in particular by lay rescuers in a residential setting.

## Notes

**Consent**: The patient’s written consent was obtained according to the COPE guidelines.

**Funding**: None declared.
